# Insured-non-insured disparity of catastrophic health expenditure in Northwest Ethiopia: a multivariate decomposition analysis

**DOI:** 10.1186/s13561-024-00533-3

**Published:** 2024-07-17

**Authors:** Yawkal Tsega, Gebeyehu Tsega, Asnakew Molla Mekonen, Tesfaye Birhane, Elsabeth Addisu, Abebe Getie, Fekade Demeke Bayou, Mulugeta Desalegn Kasaye, Natnael Kebede, Amare Muche

**Affiliations:** 1 Department of Health Systems and Management, School of Public Health, College of Medicine and Health Sciences, Wollo University, Dessie, Ethiopia; 2 Department of Health Systems Management and Health Economics, School of Public Health, College of Medicine and Health Sciences, Bahir Dar University, Bahir Dar, Ethiopia; 3 Department of Reproductive and Family Health, School of Public Health, College of Medicine and Health Sciences, Wollo University, Dessie, Ethiopia; 4 Department of Epidemiology and Biostatistics, School of Public Health, College of Medicine and Health Sciences, Wollo University, Dessie, Ethiopia; 5 Department of Health Promotion, School of Public Health, College of Medicine and Health Sciences, Wollo University, Dessie, Ethiopia; 6 Department of Pharmacy, College of Medicine and Health Sciences, Wollo University, Dessie, Ethiopia; 7 Department of Health Informatics, School of Public Health, College of Medicine and Health Sciences, Wollo University, Dessie, Ethiopia

**Keywords:** Insured-non-insured disparity, Catastrophic health expenditure, Decomposition analysis

## Abstract

**Background:**

Financial risk protection is one indicator of universal health coverage (UHC). All people should be protected from financial risks such as catastrophic health expenditures (CHE) to ensure equitable health services. Ethiopia has launched community-based health insurance (CBHI) since 2011 to protect people from financial risk. However, out-of-pocket health expenditure is a financial barriers to achieve UHC. The insured-non-insured disparity of CHE has not been well studied in Ethiopia in general and in Debre Tabor town in particular. Therefore, this study aimed to assess the disparity of CHE between insured and non-insured households and its contributing factors in Debre Tabor town.

**Methods:**

This study used the primary household survey data collected from May to June 2022 in Debre Tabor town. Data were collected from 825 household heads and analyzed using STATA version 17.0 statistical software. Logit-based multivariate decomposition analysis was conducted to determine insured-non-insured disparity of CHE. Statistical significance for all analysis was declared at a *p* < 0.05.

**Results:**

The incidence of CHE was 17.94% and 5.58% among non-insured and insured households, respectively. About 53% and 153.20% of the insured-non-insured disparities in the magnitude of CHE were due to the difference in characteristics (endowments) and the effect of characteristics (coefficients), respectively. Age of the household head between 46 and 60 years and above 60 years, divorced and widowed marital status of household head, and chronic health conditions were the explanatory variables widening the gap in the incidence of CHE. However, do not seeking traditional medicine, family size above 4, and age of household head between 31 and 45 years were the variables contribute in reducing the gap (i.e. due to endowments) in the incidence of CHE between insured and non-insured households. Moreover, the variables that contributed to the gap in the incidence of CHE due to covariate effects were age (31–45) and marital status of household head, wealth status, family size, ownership of the household, and seeking traditional medicines.

**Conclusion:**

This study revealed there is a significant disparity in the incidence of CHE between insured and non-insured households. Age, marital status and occupation of the household head, family size of household, presence of a chronically ill household member and seeking traditional medicine were significantly contributing factors for the disparity of CHE between insured and non-insured households due to endowments. The variables that contributed to the disparity in the incidence of CHE due to covariate effects were age and marital status of household head, wealth status, family size, ownership of the household, and seeking traditional medicines. Therefore, the policy makers need to emphasize in increasing the insurance coverage among households, and providing affordable health services in Ethiopia in general and Debre Tabor town in particular.

## Background

The Sustainable Development Goals (SDGs) aim to achieve universal health coverage (UHC), along with Ethiopian Health Sector Transformation Plan II (HSTP II). Financial risk protection (FRP) is one indicator of universal health coverage (UHC) and a priority area of Ethiopia as evidenced from its Health Sector Transformational Plan II (HSTP II). Lack of FRP, due to large share of out-of-pocket spending, can be measured through catastrophic health expenditure(CHE) [1–3]. Universal Health Coverage (UHC) is a crucial issue in the realm of global health policy, particularly for low- and middle-income countries (LMICs). UHC is rooted in the Sustainable Development Goals (SDGs), which strive to guarantee health and well-being for everyone, regardless of age, by the year 2030. This includes FRP and equitable access to high-quality essential healthcare services [4–6].

Households obligated to pay, direct medical, direct non-medical, indirect medical, and intangible medical costs in order to access essential health care. Direct out of pocket (OOP) healthcare expenditure is the major financial risk and cause of disparity and inequity in the provision of health care services in low-income countries such as Ethiopia [1, 2, 7–9].

The SDG indicator 3.8.2 is monitored and tracked by using the metrics of CHE and impoverishing health expenditure (IHE). When healthcare costs exceed a certain threshold (ranged from 10 to 40% of household income or expenditures), it is considered that the households experienced CHE [1, 2, 10].

Since 2000, there has been increment in the incidence of CHE worldwide. For instance, using a 10% threshold level, the incidence of CHE raised by 3.6% annually, from 571 million in 2000 to 927 million in 2015. Likewise, at the 10% threshold level, the incidence of CHE increased from 12.7% in 2015 to 13.2% in 2017. If the share of OOP health spending continues its current pace, it will continue to rise until the year 2030 [1, 2].

CHE contributes to disparities in the provision of essential quality healthcare services. The disparities are attributed to various factors like insurance status of the household, having chronic health conditions and being socially unsupported [7, 8]. This burden is directly proportional to the severity of the underlying health condition (ill individuals spend more). CHE leads the households to delay or forgo quality essential health services [2, 11, 12].

In LMICs, low health care resources and a lack of protection from financial risks led them to depend on OOP health spending. Households who rely on OOP healthcare payment and who are unable to cope with the economic burdens of illness are frequently experienced CHE which lead households lack of meeting other subsistence requirements such as food and education [4, 13].

Moreover, in low-income countries, OOP health expenditures accounts for greater than half of their total expenditure and more than one third in middle-income countries. As of World Health Organization (WHO), OOP payments push millions of households into impoverishment each year, and many of them are at risk of financial risk due to health care as well [14].

Catastrophic health expenses are concentrated among the worse off than the better off, including African countries. Inequities in accessing quality health care exist in Sub-Saharan African (SSA) countries as a result of income disparities and the level of OOP health expenditure. The percentage of households suffering by CHE has been proven to differ significantly among countries [15].

Since CHE is the most challenging and a priority area of the health sector, Ethiopian healthcare financing reform has been applied since 1998. For instance, various FRP measures such as fee waiver system and launching community based health insurance(CBHI) have been executed [16]. Though, OOP health expenditures remain a significant financial problem of households. For example, as the 8th Ethiopian Health Account (EHA), OOP health spending amounted 30% of the total health expenditure, which is too high and it is higher than the WHO recommended target, 20% [17, 18]. As a result, households often obliged to borrow money, sell their assets, reduce consumption of other basic needs to spend on healthcare services and my forgo essential healthcare services [15, 19].

Evidence, on insured-non-insured disparity in the incidence of CHE and its attributing factors among households, is critical to ensure equitable and affordable access to quality health services in Ethiopia. However, up to the best knowledge of investigators, the topic has not been well studied in Ethiopia. Therefore, the aim of this study is to assess insured-non-insured disparity and contributing factors of CHE in Debre Tabor town, Northwestern Ethiopia.

## Methods

### Study setting, design and period

The study was conducted in Debre Tabor town, the capital of South Gondar zone. There are 84,384 people living in the town, making up 19,624 households, in which 19,898 are reproductive aged women and 10,868 are children between the ages of 6 and 59 months. The town is 108.6 km far from the east of Bahir Dar, the Amhara state capital. Moreover, it has 6 kebeles(the smallest administrative structure in Ethiopia), one comprehensive specialized hospital and four health centers [20]. Community based cross-sectional study design was conducted from May 24/2022 to June 17/2022.

### Populations and eligibility criteria

All households in Debre Tabor town were source populations and households in randomly selected kebeles, the smallest administrative unit in Ethiopia, such as kebele 01, kebele 03 and kebele 04 were the study populations. All household heads lived in Debre Tabor town for 6 months and above were included in the study and household heads unable to respond due to critical illness, unable to speak and listen were excluded from the study.

### Variables and measurements

#### Outcome variable

The CHE was the outcome variable. Wagstaff and van Doorslaer approach was used to measure CHE. This approach considers CHE when the share of household’s health expenditure from total household expenditure/income/nonfood expenditure exceeds a certain threshold level. The choice of the threshold level is arbitrary and varied from 10 to 40% [21]. The outcome variable was CHE and classified dichotomously as "Yes/No". Households with their health expenditure exceeds 10% of their total expenditure were categorized as they experienced CHE (i.e., Yes, leveled as “1”) otherwise No (leveled as “0”).

To measure CHE, annual average health expenditure for each household was estimated by summing up all self-reported healthcare expenditures from May 2021 to May 24/2022, 12 months back. All the expenditures for transportation, cafeteria and lodging were summed up based on the self-reported number of household members having history of illness and amount of money they incurred. Data on indirect costs covered in this study included lost days (absenteeism), premature death and early retirements due to healthcare expenditure both for the patient and caregiver as per human capital approach.

#### Stratifying variable

The equity stratifying variable was insurance status of the households which was categorized as insured (leveled as 0) and non-insured (leveled as 1) if the household heads reported the household enrolled for CBHI and not enrolled for it, respectively.

#### Explanatory variables

The explanatory variables were grouped to three categories such as sociodemographic variables (sex, age, and marital status of household head, presence of under five children, and family size), socio-economic characteristics (wealth status, occupation of household head, ownership of household), and health profile of the household (referral history, chronic health condition, and seeking traditional medicine). Wealth index was constructed using principal component analysis (PCA) by STATA version 17.0 statistical software. The constructing variable scores were derived using PCA in that; 35 wealth status assessing variables from sanitation, housing condition, water source and household durable assets were computed. Variables having frequency of greater than 95% and less than 5% were excluded. In PCA output of correlation matrix, values < 0.1 and > 0.9 were removed from the analysis. After all, 12 variables were used to construct the wealth index. The first component of the composite variables was used to estimate wealth status of households and ranked in ascending order [7].

### Operational definition

#### Household

 A person or group of persons, whether or not they are related, who normally live together in the same housing unit or group of housing units, and who have common cooking arrangements [22].

#### Catastrophic health expenditure (CHE)

 Spending greater than 10% of household’s reported total expenditure for healthcare service [1–3].

#### Healthcare expenditure

 The total household expenditure for healthcare, which included direct medical, direct non-medical and indirect costs [2].

#### Wealth index

The composite measure of cumulative living standard of the household. It was measured by 35 different variables [17, 23].

#### Chronic health condition

Is a human health condition or disease that is persistent or otherwise long-lasting in its effects or a disease that comes with time. The term chronic was used when the course of the disease lasts for more than three months in this study [24].

#### Insurance status

In this study means community based health insurance enrolment status (CBHI) [25].

### Data sources

This study used the primary household survey dataset collected by trained Public Health professionals from May to June 2022 in three randomly selected kebeles of Debre Tabor town.

### Sample size, sampling technique and procedures

Single population proportion formula was used to estimate the sample size by using the assumptions of proportion 50% of CHE at 10% threshold level, confidence level of 95%, degree of precision 5%, non-response rate of 10% and design effect of 2. It was determined using the following 

formula.

$$\mathrm n=\frac{\mathrm Z\;\left(\mathrm\alpha/2\right)^2\ast\mathrm P\;\left(1-\mathrm q\right)}{\mathrm d^2}$$

Where *P* = 50%

$$\mathrm d=0.05\;(\mathrm{degree}\;\mathrm{of}\;\mathrm{precision})\;\mathrm{and}\;\mathrm Z\;\mathrm\alpha/2\;\mathrm{at}\;95\%\;\mathrm{confidence}\;\mathrm{level}\;=1.96$$

By taking the above values, the sample size was.

$$\mathrm n=\frac{\left(1.96\right)^{2\ast}\;\left(0.5\right)\;\left(1-0.5\right)=384;\;384\ast10\%\mathrm{NRR}\;+384\;=423\ast2=846}{\left(0.05\right)^2}$$

**Fig. 1:**
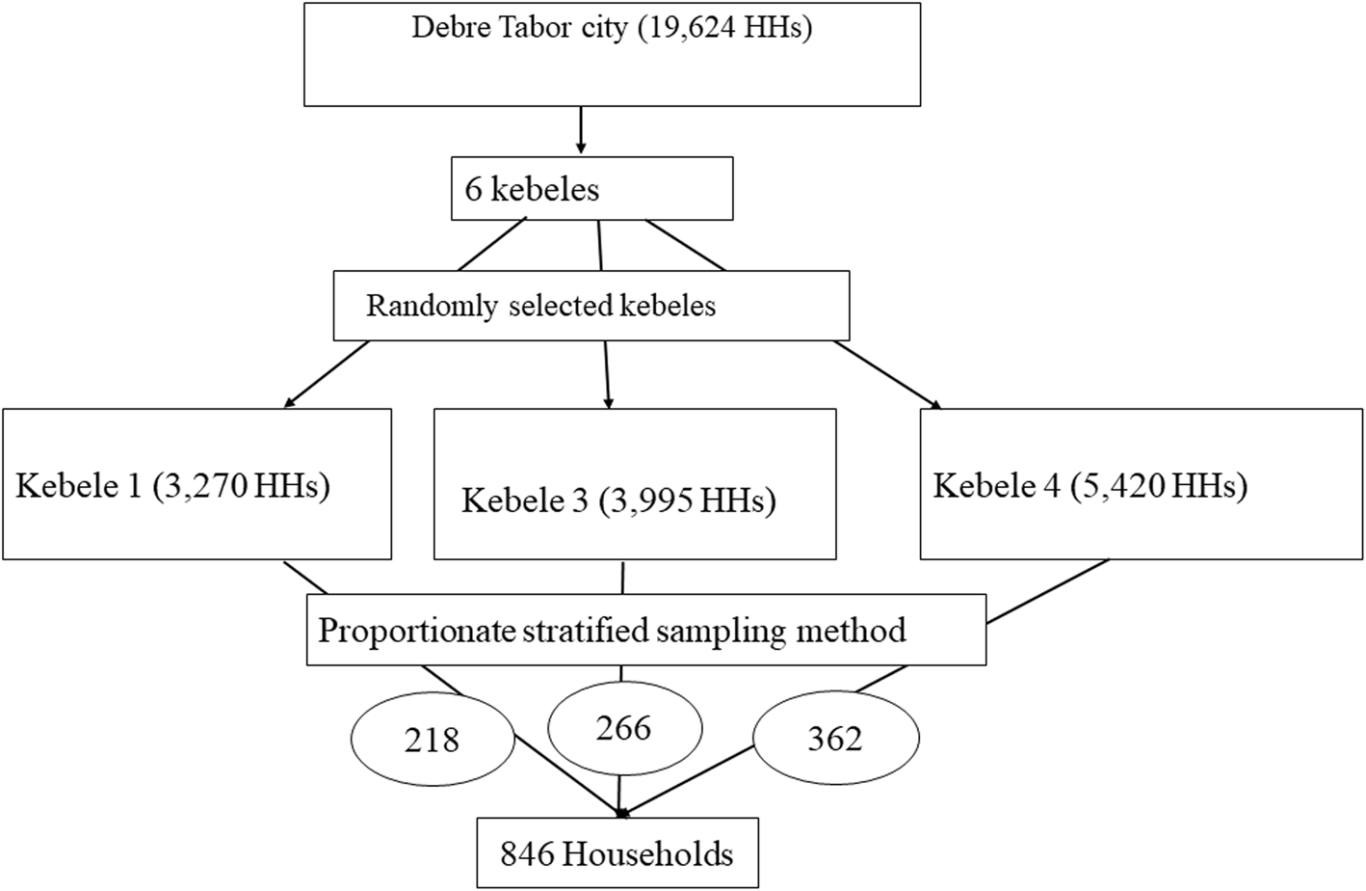
Sampling procedure for the study of insured-non-insured disparity of Catastrophic Health Expenditure, Debre Tabor, South Gondar zone, northwest, 2022

Debre Tabor town has six kebeles. Of which, we randomly selected three Kebeles (Kebele 01, Kebele 03 and Kebele 04). The sample size was proportionally allocated for each selected kebeles and 846 households were selected using systematic random sampling method in each of the three randomly selected kebeles and included for multivariate decomposition analysis (Fig. 1).

### Survey tools and data quality assurance

Structured questionnaire was used and developed after reviewing various relevant literatures. The questionnaire was prepared in English first and translated to Amharic (local language) for better understanding with respondents. Three data collectors with educational level of bachelor of degree (public health graduates) and one supervisors (MPH) were employed. Three days training was given for data collectors on the overall picture of data collection process. Before actual data collection, pretesting on 5% of the sample size was done in Woreta town. Close supervision of data collectors was done and data were checked for its completeness on daily basis [7].

### Data management and analysis

#### Descriptive analysis and chi-square test

Data were entered into EpiData version 4.6 and exported to Stata version 17 for analysis. Descriptive statistics (frequency, percent) were analyzed. Before conducting the multivariate decomposition analysis, chi-square test was done to check whether explanatory variables have significant association with the outcome variable (i.e. CHE).

#### Multivariate decomposition analysis

About 10 variables were selected based on their association with CHE in chi-square test. Multivariate decomposition analysis was done to estimate the disparity in the incidence of CHE between insured and non-insured households and its attributing factors of the disparity due to endowments and coefficients [26]. The logit based multivariate decomposition analysis model was used to decompose the observed difference in the incidence of CHE into two components. The first component was due to variation in composition (endowment) of characteristics across insured and non-insured households and second $\underbrace{\mathrm{component}\;\mathrm{was}\;\mathrm{due}\;\mathrm{to}}_{\mathrm E}$ the $\underbrace{\mathrm{effect}\;\mathrm{of}\;\mathrm{characteristics}}_{\mathrm C}$ (coefficient). Thus, the disparities in outcome (i.e. incidence of CHE) were due to either the differences in the composition of characteristics (endowment) or in the effect of characteristics (coefficient), or the interaction of the two components (endowment and coefficient). The model of multivariate decomposition analysis can be determine using the following mathematical formula assuming insured and non-insured households were called A and B, respectively. For logistic regression, the logit or log-odds of CHE can be split into two parts as shown in the Eq. 1 below:

$$logit\left(A\right)-logit\left(B\right)=F\left(X_A\beta_A\right)-F\left(X_B\beta_B\right)$$

1 $$=\left[F\left(X_A\beta_A\right)-F\left(X_B\beta_A\right)\right]+\left[F\left(X_B\beta_A\right)-F\left(X_B\beta_B\right)\right]$$

Where: E represents the disparity due to endowments explained by characteristics and C represents the disparity due to coefficients (effect of characteristics), the unexplained part. The user written mvdcmp Stata command was used for multivariate decomposition analysis.

## Results

### Socioeconomic characteristics and health profile of respondents

About 825 households participated in the study, making the response rate 97.52%. Moreover, about 574(69.58%) of the households were male headed and 343(41.58%) of the household heads were found in their age with a range of 31–45 years. The mean age of the household heads was 43.37 years with a standard deviation of 14.28 (43.37 ± 14.28). The proportion of households experiencing catastrophic health expenditure was nearly 30% and about 23.5% of the households were insured for health (i.e. enrolled for community based health insurance). Regarding occupation of household heads and wealth status of households, 46.67% were government employed and 21.70% of the households were categorized as richer respectively (Table 1).

**Table 1:** Descriptive results of the study on insured-non-insured disparity in the incidence of catastrophic health expenditure, Debre Tabor, South Gondar zone, northwest Ethiopia, 2022

Variables	Category	Frequency	Percent (%)
Sex of household head	Male	574	69.58
	Female	251	30.42
Age of household head	< = 30	191	23.15
	31–45	343	41.58
	46–60	182	22.06
	> 60	109	13.21
Marital status of household head	Single	55	6.67
	Married	573	69.45
	Separated	28	3.39
	Divorced	74	8.97
	Widowed	95	11.52
Ownership of the house	Private	521	63.15
	Rent	304	36.85
Occupation	Self employed	399	48.36
	Government employed	385	46.67
	Private sectors^3^	41	4.97
Family size	<= 4	628	76.12
	> 4	197	23.88
Presence of U5 Children	Yes	261	31.64
	No	564	68.36
Wealth status	Poorest	163	19.76
	Poorer	160	19.39
	Middle	161	19.52
	Richer	179	21.70
	Richest	162	19.64
Chronic health conditions	Yes	249	30.18
	No	576	69.82
Seeking traditional medicine	Yes	178	21.58
	No	647	78.42
Referral history(n = 695)	Yes	41	5.90
	No	654	94.10
Catastrophic Health Expenditure	Yes	247	29.94
	No	578	70.06
Insurance status	Insured	194	23.52
	Non-insured	631	76.48

^3^cleaning workers, guards and waiters, and non-governmental organization workers, U5: under five

### Association of catastrophic health expenditure with explanatory variables

About 187(22.67%) male headed, 148(17.94%) non-insured and 46(5.58%) insured households experienced CHES. Moreover, almost all (~ 93%) of the households having referral history one year back to the survey have experienced CHE (Table 2).

**Table 2:** Association of explanatory variables with catastrophic health expenditure and insurance status, Debre Tabor, South Gondar zone, Northwest Ethiopia, 2022

		CHE		Insurance status	
Variables	Category	Yes (n (%))	No (n (%))	Insured (n (%))	Non-insured (n (%))
Sex of HH head	Male	187(22.67)	387(46.91)	127(15.39)	447(54.18)
	Female	60(7.27)	191(23.15)	67(8.12)	184(22.30)
Age of Household head	< = 30	27(3.27)	164(19.88)	33(4.00)	158(19.15)
	31–45	93(11.27)	250(30.30)	73(8.85)	270(32.73)
	46–60	69(8.36)	113(13.70)	56(6.79)	126(15.27)
	> 60	58(7.03)	51(6.18)	32(3.88)	77(9.33)
Marital status of household head	Single	6(0.73)	49(5.94)	18(2.18)	37(4.48)
	Married	170(20.61)	403(48.85)	116(14.06)	457(55.39)
	Separated	5(0.61)	23(2.79)	5(0.61)	23(2.79)
	Divorced	28(3.39)	46(5.58)	20(2.42)	54(6.55)
	Widowed	38(4.61)	57(6.91)	35(4.24)	60(7.27)
Presence of U5C	Yes	56(6.79)	205(24.85)	61(7.39)	200(24.24)
	No	191(23.15)	373(45.21)	133(16.12)	431(52.24)
Family size	< = 4	175(21.21)	453(54.91)	143(17.33)	485(58.79)
	> 4	72(8.73)	125(15.15)	51(6.18)	146(17.70)
Occupation	Self employed	99(12.00)	300(36.36)	158(19.15)	241(29.21)
	Gov’t employed	130(15.76)	255(30.91)	19(2.30)	366(44.36)
	Private sectors	18(2.18)	23(2.79)	17(2.06)	24(2.91)
Wealth status	Poorest	39(4.73)	124(15.03)	67(8.12)	96(11.64)
	Poorer	34(4.12)	126(15.27)	52(6.30)	108(13.09)
	Middle	46(5.58)	115(13.94)	29(3.52)	132(16.00)
	Richer	78(9.45)	101(12.24)	26(3.15)	153(18.55)
	Richest	50(6.06)	112(23.58)	20(2.42)	142(17.21)
Ownership of the house	Private	197(23.88)	324(39.27)	125(15.15)	396(48.00)
	Rent	50(6.06)	254(30.79)	69(8.36)	235(28.48)
Chronic health conditions	Yes	155(18.79)	94(11.39)	70(8.48)	179(21.70)
	No	92(11.15)	484(58.67)	124(15.03)	452(54.79)
Seeking traditional medicine	Yes	77(9.33)	101(12.24)	35(4.24)	143(17.33)
	No	170(20.61)	477(57.82)	159(19.27)	488(59.15)
Referral history(*n* = 695)	Yes	38(5.47)	3(0.43)	6(0.86)	35(5.04)
	No	209(30.07)	445(64.03)	154(22.16)	500(71.94)
Insurance status	Insured	46(5.58)	201(24.36)		
	Non-insured	148(17.94)	430(52.12)		

### Multivariate decomposition analysis

The overall decomposition result showed that there is a significant disparity in the incidence of catastrophic health expenditure between insured and non-insured households (β: 0.08, *p* < 0.003). The decomposition analysis model was done and had taken into an account the differences in the composition of characteristics (E) and the differences due to the effect of characteristics(C). A positive coefficients indicates the expected reduction the gap and negative coefficients suggested the expected widening of the gap in the incidence of CHE between insured and non-insured households (Table 3).

**Table 3:** Detailed decomposition of Catastrophic Health Expenditure by insurance status among households in Debre Tabor, South Gondar zone, Northwest Ethiopia, 2022

Decomposition	Coefficient with 95% CI			Percent Explained	*P-*value
Row difference(R)	0.081429(0.027, 0.13527)			100	**0.003**
Explained(E)	-0.04332(-0.0806, -0.00609)			**-53.20**	**0.023**
Unexplained(C)	0.12475(0.05857, 0.19093)			153.20	**0.000**
**Endowment (Explained component) = Difference in characteristics (E)**					
		Coefficient with 95% CI		Percent	*P* value
Sex of HH head	Male	1		1	
	Female	0.0040208(-0.0021219, 0.010164)		4.9379	0.200
Age of Household head	<= 30	1		1	
	31–45	0.0079854(0.0023114, 0.013659)		9.8067	**0.006**
	46–60	-0.01383(-0.026734, -0.00092733)		-16.985	**0.036**
	> 60	-0.013954(-0.021827,-0.0060814)		-17.137	**0.001**
Marital status of household head	Single	1		1	
	Married	0.028293(-0.0044437, 0 .06103)		34.746	0.090
	Separated	0.0025887(-0.0007143, 0.0058917		3.1791	0.124
	Divorced	-0.006879(-09.012034, -0.001725)		-8.4481	**0.009**
	Widowed	-0.028591(-0.053218, -0.0039633)		-35.111	**0.023**
Presence of U5C	Yes	-0.0000932(-0.0003359, 0.00015)		-0.11443	0.452
	No	1		1	
Family size	<= 4	1		1	
	> 4	0.003998(0.00052148, 0.0074744)		4.9098	**0.024**
Occupation	Self employed	1		1	
	Gov’t employed	0.011677(-0.033091, 0.056444)		14.34	0.609
	Private employed	-0.025183(-0.037005, -0.01336)		-30.926	**0.000**
Wealth status	Poorest	1		1	
	Poorer	-0.00016085(-0.01398, 0.013658)		-0.19754	0.982
	Middle	0.00098(-0.0071452, 0.0091081)		1.2053	0.813
	Richer	0.0052064(-0.0095939, 0.020007)		6.3938	0.491
	Richest	0.0082929(-0.0083175, 0.024903)		10.184	0.328
Ownership of the house	Private	1		1	
	Rent	0.00061449(-0.001129, 0.002358)		0.75464	0.490
Chronic health conditions	Yes	1		1	
	No	-0.036467(-0.050642, -0.022292)		-44.784	**0.000**
Seeking traditional medicine	Yes	1		1	
	No	0.0081797(0.003026, 0 .013334)		10.045	**0.002**
**Unexplained (Due to difference in coefficients (C))**					
Sex of HH head	Male	1		1	
	Female	0.017436(-0.043064, 0.077936)		21.413	0.572
Age of Household head	< = 30	1		1	
	31–45	0.048597(-0.023644, 0.12084)		59.681	0.187
	46–60	0.054605(-0.0061614, 0.11537)		67.059	0.078
	> 60	0.048753(0.0075756, 0.089931)		59.872	**0.020**
Marital status of household head	Single	1		1	
	Married	-0.05287(-0.18134, 0.075595)		-64.928	0.420
	Separated	-0.004668(-0.015486, 0.0061504)		-5.7322	0.398
	Divorced	-0.033056(-0.058998, -0.007114)		-40.595	**0.013**
	Widowed	-0.024426(-0.068075, 0 .019223)		-29.997	0.273
Presence of U5C	Yes	-0.010797(-0.046225, 0.024631)		-13.259	0.550
	No	1		1	
Family size	< = 4	1		1	
	> 4	-0.057794(-0.094269, -0.021318)		-70.975	**0.002**
Occupation	Self employed	1		1	
	Gov’t employed	-0.005239(-0.020016, 0.0095378)		-6.4341	0.487
	Private sectors	0.010788(-0.0075941, 0.02917)		13.249	0.250
Wealth status	Poorest	1		1	
	Poorer	-0.017668(-0.055656, 0.020321)		-21.697	0.362
	Middle	-0.019503(-0.040229, 0.0012234)		-23.951	0.065
	Richer	-0.022883(-0.042226, -0.0035391)		-28.101	**0.020**
	Richest	0.00013849(-0.018173, 0.01845)		0.17008	0.988
Ownership of the house	Private	1		1	
	Rent	0.13244(0.089343, 0 .17554)		162.65	**0.000**
Chronic health conditions	Yes	1		1	
	No	-0.018035(-0.097643, 0.061573)		-22.148	0.657
Seeking traditional medicine	Yes	1		1	
	No	0.11367(0.02136, 0.20598)		139.6	**0.016**

About 53.20% (*p* < 0.023) of the observed disparity in the incidence of CHE were explained by the differences in distribution of characteristics (i.e. explained part) between insured and non-insured households. Moreover, the majority of the gap in the incidence of CHE was explained by presence of chronic health conditions among household members differences between insured and non-insured households, absence of chronically ill household member(-44.78%) contributed for widening of the gap. That means if the composition of not chronically ill members among non-insured households were equalized to the insured households, the gap in the incidence of CHE would expected to widen the gap by 44.78%. Age of household head ranged 31–45 years (9.81%), large household size (> 4) (4.91%), and not seeking traditional medicine(10.05%) were factors helping to achieve narrowing of the insured-non-insured gap in the incidence of CHE if these covariates distribution equalized to the level of insured households for non-insured households as well (Table 3).

However, age of household head ranged from 46–60(-17.00%) and > 60 years (-17.14%), household head being divorced (-8.45%) and widowed (-35.11%) in marital status, and being the private organization employed (-30.93%) were factors contributing for the widening of the gap in the incidence CHE between insured and non-insured households. That is, if insured households were not protected from financial risks of healthcare as the same rate as non-insured households, the gap between insured and non-insured households in CHE would be expected to increase by the percentages with their respective covariates, mentioned above (Table 3).

Additionally, we found that differences in the effects of characteristics account for 153.20% (*p* < 0.003) of the observed insured-non-insured households difference in the incidence of CHE. Moreover, the difference in the effect of the household being rented (162.65%), household head age > 60 years(59.87%) and not seeking traditional medicine (139.60%) contributes for narrowing the gap of incidence of CHE between insured and non-insured. Whereas, the difference in the effect of marital status of household head being divorced, larger household size (family size > 4) and richer wealth status of the household were the factors contribute for widening of insured-non-insured incidence of CHE by 40.60%, 70.98%, and 28.10%(Table 3).

## Discussion

This study aimed to assess insured-non-insured disparity in the incidence of CHE, including the contributing factors for the difference among households in Debre Tabor. The multivariate decomposition analysis, in the context of the insured-non-insured gap, helps by breaking down the complex issue into simpler components. It identifies and quantifies the contribution of key factors to the observed gap. This detailed understanding not only guides effective policy interventions but also enables comparisons across different groups or over time, revealing trends or disparities [27].

The result of this study revealed that incidence of CHE among non-insured households was higher (i.e. overall(incidence: ~ 30%), non-insured(incidence: 17.94%) and insured(5.58%), which implies that non-insured households are more vulnerable to financial risk of health care expenditures almost triple times. Moreover, the finding suggested that CBHI, which has been implemented in Ethiopia since 2011, is paramount to protect households from financial risks of health care expenditure. This implication is also supported by different documented evidences like the studies conducted in Debre Tabor town in the same period and northeast Ethiopia in 2017 [7, 8].

Moreover, the study indicated that about 53.20% of the disparities were due to distributional (endowment) differences and 153.20% disparities were due to difference in the effect of characteristics. The study findings have significant implications for healthcare providers, policymakers, and stakeholders. These findings suggest that interventions designed to address both the distributional differences and the effects of characteristics, such as financial assistance programs or health education initiatives, could be effective in reducing these disparities. Moreover, the larger impact of the effects of characteristics implies that policies aimed at reducing these effects, such as increasing access to insurance or improving the affordability of healthcare, could have a more significant impact on reducing disparities than policies aimed solely at addressing distributional differences. Therefore, these findings provide valuable insights that can inform the development of effective policies and interventions to reduce disparities in the incidence of catastrophic health expenditure [28, 29].

The study's findings also revealed that several factors that contribute to the gap in the incidence of CHE between insured and non-insured households. For instance, the age of the household head (31–45 years), large household size, and not seeking traditional medicine were found to narrow the gap if these covariates were equalized to the level of insured households. This suggests that policies designed to equalize these factors could potentially reduce the incidence of CHE. On the other hand, factors such as the age of the household head (46–60 and > 60 years), marital status of the household head (divorced and widowed), and employment in a private organization were found to widen the gap. These findings underscore the complexity of the issue and the need for multifaceted policy interventions that address both the factors that narrow and widen the gap in catastrophic health expenditure [30].

The study found that certain factors significantly reduce the disparity in CHE between insured and non-insured households. These factors include the household being privately owned (162.65%), the household head being 30 years old or younger (59.87%), and the household not seeking to traditional medicine (139.60%). If non-insured households transitioned from rented to privately owned(if the household is private), if the age of non-insured household heads reduced to 30 years or less, and if members of insured households opted for traditional medicine like their counterparts in insured households, the gap in CHE would be expected to decrease by the mentioned percentages. The policy implications of these findings suggest that if the government could facilitate all households to become privately owned, the disparity in CHE could potentially be reduced. Similarly, if non-insured households refrained from seeking traditional medicine the gap in CHE could also be expected to decrease [31, 32].

However, the study also revealed that the effect of marital status of household head being divorced, larger household size (family size > 4) and richer wealth status of the household were the factors responsible for widening of insured-non-insured incidence of catastrophic health expenditure gap. This implies that being divorced in marital status having larger household size are the risk factors to increase financial risks of health care and increasing the gap between insured and non-insured households.

### Limitation of the study

Recall bias was the primary study drawback. Although steps have been taken, such as triangulating self-reported health spending with the recipients(their payment bills for health care) to lessen recall bias, it is still a drawback of this study.

## Conclusions and recommendations

The significant disparity exists in catastrophic health expenditure between insured and non-insured households. The majority of this discrepancy is attributed to covariate effects. Factors such as the age, marital status, and occupation of the household head, the size of the family, the presence of a chronically ill member in the household, and the use of traditional medicine contribute to this disparity due to differences in endowments. The variables that contribute to the differences in catastrophic health expenditure coefficients between insured and non-insured households include the age and marital status of the household head, the wealth status, family size, ownership of the household, and the use of traditional medicine. This suggests that the government should prioritize addressing covariate effects over distributional differences to protect households from the financial risks associated with healthcare expenditure.

## Data Availability

All relevant data are found in the manuscript.

## Abbreviations

CBHI: Community Based Health Insurance
CHE: Catastrophic Health Expenditure
EHA: Ethiopian Health Account
HHs: Households
HSTP: Health Sector Transformation Plan
LMICs: Low and Middle Income Countries
OOP: Out of pocket
SDG: Sustainable Development Goals
SSA: Sub Saharan
UHC: Universal Health Coverage
FRP: Financial Risk Protection
WHO: World Health Organization
